# Delayed surgical strategy for type A aortic dissection associated with acute hepatitis C cryoglobulinaemia

**DOI:** 10.1093/jscr/rjac480

**Published:** 2022-10-27

**Authors:** Amber Ahmed-Issap, Lognathen Balacumaraswami

**Affiliations:** Department of Cardiothoracic Surgery, Royal Stoke Hospital, University Hospitals of North Midlands NHS Trust, Stoke-on-Trent, UK; Department of Cardiothoracic Surgery, Royal Stoke Hospital, University Hospitals of North Midlands NHS Trust, Stoke-on-Trent, UK

**Keywords:** aortic dissection, hepatitis, delayed treatment, type A

## Abstract

An aortic dissection is a condition resulting from a tunica intima tear of the aortic wall creating a ‘false lumen’. An acute Stanford type A (involves the aortic arch and/or ascending aorta) aortic dissection requires emergency surgical repair. To our knowledge, we report the first case in the literature where the treatment for an acute type A aortic dissection was intentionally delayed. This was decided following a multidisciplinary team discussion where it was agreed that the patient’s active hepatitis C infection should be treated prior to surgery. The patient re-presented to the hospital 4 months later with acute dyspnoea and orthopnoea where he was diagnosed with an acute-on-chronic type A aortic dissection with trachea compression. This was successfully treated with emergency surgery. However, the patient suffered residual dyspnoea, likely due to phrenic nerve injury demonstrating the impact of untreated aortic arch distension on the neighbouring trachea and phrenic nerve.

## INTRODUCTION

An aortic dissection is caused by a tear in the tunica intima layer creating a ‘false’ lumen resulting in reduced perfusion to the end-organs [1]. Patients classically present with severe, tearing chest pain [2]. Stanford type A aortic dissections (affecting the aortic arch and/or ascending aorta) carry a very high mortality rate with death occurring in 50% of cases within 48 hours [3]. The risk of mortality increases incrementally without intervention. It is a Class I indication for emergency life-saving surgery in order to reduce the mortality risk [1].

## CASE REPORT

**Figure 1:**
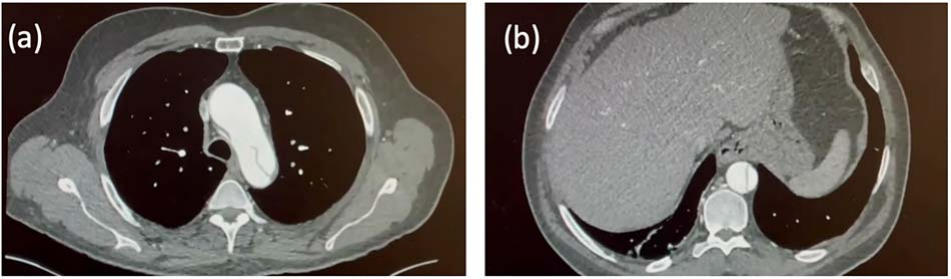
Initial CT aortogram. Aortic dissection originating from the origin of left subclavian artery with the ascending aorta measuring 45 mm (**a**). The dissection extends to involve the descending aorta and abdominal aorta (**b**).

A 54-year-old gentleman with a 30 pack-year history presented to the Emergency Department of a district hospital with sudden, severe upper abdominal pain, per rectum bleeding and vomiting. A computed tomography (CT) scan not only showed bowel ischaemia but also revealed an aortic dissection extending from the lower thoracic aorta into the superior mesenteric artery and bilateral common iliac arteries. An urgent CT aortogram demonstrated that the dissection extended from the left subclavian artery origin and involved the entire descending and abdominal aorta. The patient was diagnosed with a Stanford type B aortic dissection. The ascending aorta measured 45 mm (Fig. 1). The patient was haemodynamically stable and managed conservatively. He was transferred to a tertiary centre hospital for further care.

**Figure 2:**
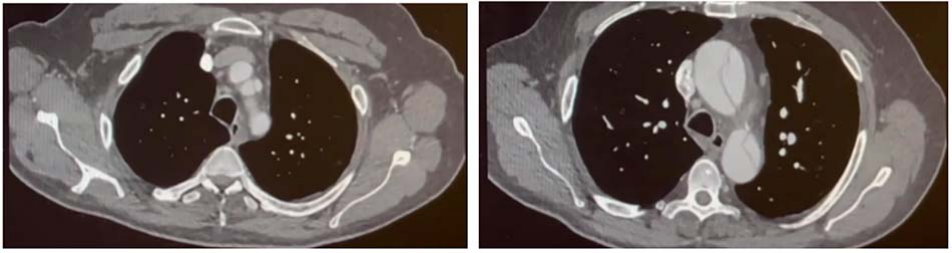
Follow-up CT aortogram. Aortic dissection extending into the left common carotid artery and involving the left subclavian artery without compromising the lumen. The ascending aorta has further dilated from Fig. 1 to now measure ~51 mm.

Seven days later, a repeat CT aorta angiogram showed that the dissection had extended into the left common carotid artery (Fig. 2). The ascending aorta had further dilated (51 × 54 mm) due to retrograde dissection extending into the aortic root. The patient was now diagnosed with a Stanford type A aortic dissection involving the aortic root, ascending aorta and aortic arch. He was scheduled for complex emergency proximal thoracic aortic reconstructive surgery. The patient’s bloodwork was sent for routine pre-operative testing.

Blood tests revealed the presence of an undiscovered active hepatitis C virus-related cryoglobulinaemia. This was related to his previous intravenous drug use. Since the patient was able to survive through the initial period following the discovery of the type A aortic dissection, it was decided that the surgery would be delayed until the hepatitis C viral load was reduced. He was commenced on a 12-week treatment of Epclusa.

**Figure 3:**
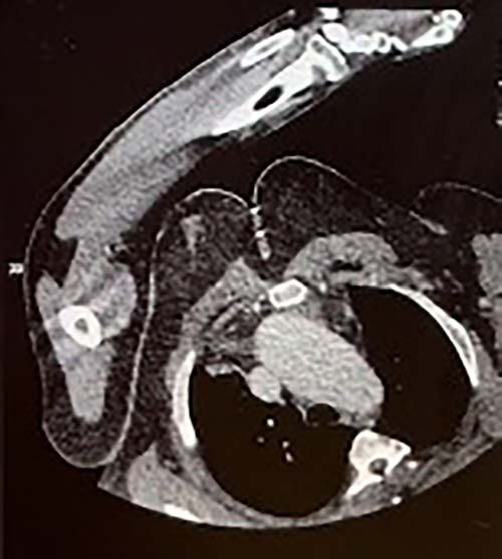
CT aorta thorax on patient’s re-admission four months later. Ascending aortic aneurysm has increased in size (measuring 60 × 61 mm) compressing the trachea.

Four months later, the patient developed dyspnoea and orthopnoea. He was also found to be positive for COVID-19. He was clinically well and his hepatitis C viral load was undetectable. A CT aorta-thorax demonstrated that the ascending aorta had further dilated (60 × 61 mm). This aneurysm compressed the trachea resulting in his acute respiratory symptoms (Fig. 3). The patient was diagnosed with an acute-on-chronic type A aortic dissection with mild–moderate aortic regurgitation. Emergency thoracic aortic surgery was performed with remodelling of the sinotubular junction with a prosthetic graft and aortic valve repair. The patient had a long post-operative recovery phase complicated primarily due to his COVID-19 diagnosis. He was discharged 43 days after his surgery.

The patient received follow-up care by the Vascular and Cardiothoracic team. The stable type B aortic dissection only required medical management. Five months following discharge, the patient attended clinic complaining of persistent dyspnoea. A chest x-ray revealed a raised diaphragm. The dyspnoea was thought to be likely due to phrenic nerve damage from traction injury that had occurred from acute aortic arch distension prior to surgery. This gradually resolved without treatment over the following months.

## DISCUSSION

Aortic dissections are often initially misdiagnosed when presenting in the emergency setting [4]. It is reported that misdiagnosis occurs in 31–39% of cases [4, 5]. Research indicates that the incidence of acute type A aortic dissections is between 2.1 and 16.3 per 100 000 people [6]. Chronic type A aortic dissections are reported to be even more rare [7]. The decision to intentionally delay treatment for a diagnosed acute type A aortic dissection, as in this case, has not yet been reported in the literature.

The patient had numerous risk factors associated with the development of an aortic dissection [1] including an extensive smoking history and a history of intravenous drug use. The patient was also diagnosed with hepatitis C virus-related cryoglobulinaemia. Fakunaga *et al.* [8] have reported a case of aortic dissection caused by aortitis associated with hepatitis C-related cryoglobulinaemia. The authors demonstrated that inflammatory cells and immunoglobulins were deposited in the vascular endothelium leading to aortic wall instability. We postulate that this aortic dissection could be attributed to multiple mechanisms including hepatitis C-induced cryoglobulinaemia.

The patient’s blood tests indicated that he had liver disease due to his viraemia. These patients are at a particular high risk of morbidity and mortality following surgery [9]. This patient had delayed treatment for his type A aortic dissection as it was necessary to first treat his acute viraemia. This decision was not taken lightly—a multidisciplinary team conducted a risk—benefit analysis and consequently decided that the hepatitis C infection must be treated prior to undertaking emergency aortic surgery since the post-operative morbidity and mortality associated with the hepatitis C infection outweighed the risks of an untreated stable type A aortic dissection.

Following successful high-risk surgery, the patient reported persistent dyspnoea. A chest x-ray revealed a raised diaphragm five months following surgery. Diaphragmatic weakness was suspected. Anatomically, the left phrenic nerve passes anteriorly over the medial part of the left subclavian artery and distal aortic arch. Diaphragmatic weakness may be attributed to prolonged traction of the phrenic nerve from the distended aorta causing neuropraxia. Over the next few months, the patient now reports less dyspnoea and the neuropraxia is potentially anticipated to resolve with time.

Type A aortic dissections remain to have an extremely high associated mortality rate, which means that patients are rushed into surgery soon after diagnosis. This report demonstrates an interesting case of delayed emergency treatment of a diagnosed type A aortic dissection despite the associated mortality risk. Although this patient survived this ordeal, we believe that each case should be assessed on its own merit since this is a very unique scenario and aortic dissection treatment should generally not be delayed due to the increased mortality risk.

## CONFLICT OF INTEREST STATEMENT

None declared.

## FUNDING

None.
